# Natural waxes from plant and animal origin as dielectrics for low-voltage organic field effect transistors

**DOI:** 10.1039/d5tc01419k

**Published:** 2025-05-29

**Authors:** Cristian Vlad Irimia, Cigdem Yumusak, Yasin Kanbur, Corina Schimanofsky, Boyuan Ban, Martin Ciganek, Petr Sedlacek, Jozef Krajcovic, Rosarita D’Orsi, Alessandra Operamolla, Oliver Brüggemann, Yolanda Salinas, Andreas Petritz, Barbara Stadlober, Rahul Mourya, Christian Teichert, Heinz Langhals, Niyazi Serdar Sariciftci, Mihai Irimia-Vladu

**Affiliations:** a Linz Institute for Organic Solar Cells (LIOS), Institute of Physical Chemistry, Johannes Kepler University Linz Altenberger Str. 69 4040 Linz Austria mihai.irimia-vladu@jku.at; b Department of Chemistry, Karabük University, Baliklarkayasi Mevkii Karabük 78050 Turkey; c Chair of Physics, Department of Physics, Mechanics, and Electrical Engineering, Montanuniversität Leoben Franz Josef Str. 18 8700 Leoben Austria; d Institute of Solid-State Physics, Hefei Institute of Physical Science, Key Lab of Photovoltaic and Energy Conservation Materials Hefei China; e Brno University of Technology, Faculty of Chemistry Purkyňova 464/118 612 00 Brno Czech Republic; f Department of Chemistry and Industrial Chemistry, University of Pisa via Giuseppe Moruzzi 13 54126 Pisa Italy; g Institute of Polymer Chemistry, Johannes Kepler University Linz Altenberger Str. 69 Linz 4040 Austria; h IMC Krems University of Applied Sciences, Institute of Applied Chemistry Piaristengasse 1 3500 Krems Austria; i Joanneum Research Materials, Institute for Surface Technologies and Photonics Franz-Pichler Str. Nr. 30 Weiz 8169 Austria; j A.F. Suter & Company Ltd, Compass House, Eastways Industrial Estate Witham Essex CM8 3YQ UK

## Abstract

We demonstrate in this work the practical use of naturally extracted waxes of plant and animal origin, *i.e.*, beeswax, carnauba wax, rice bran wax, lanolin wax, and two shellac waxes as dielectrics in organic field effect transistors (OFETs). We present a thorough characterization of their material properties, processability and film forming characteristic, surface characterization, dielectric investigation and the fabrication of field effect transistors with two classic organic semiconductors, *i.e.*, pentacene and fullerene C_60_. We show that operating voltages as low as 1 V are possible for all the OFETs using blade coating as fabrication method of waxes solubilized in their appropriate solvent, chloroform or *n*-octane. Although in general difficult to process in thin films, we demonstrate in this work the practical applicability of these natural waxes for electronics fabrication.

## Abstract 2

Six naturally occurring waxes of plant and animal origin are demonstrated to be excellent dielectric materials for organic field effect transistors operating at low voltage in the frame of sustainable electronics development.

## Graphical abstract

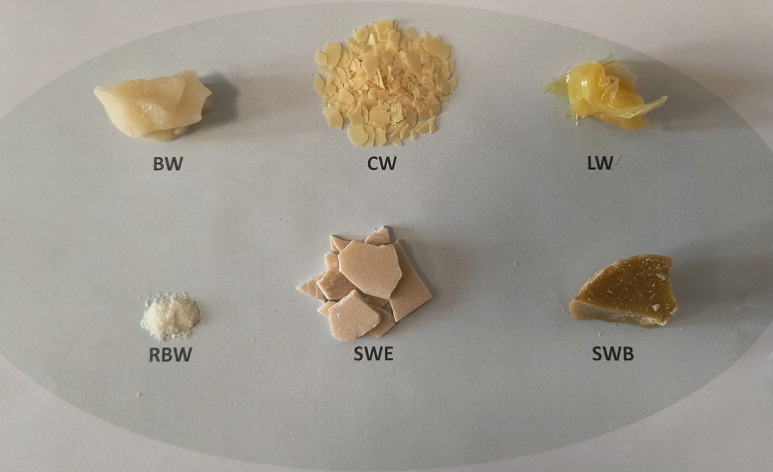

## 1. Introduction

A great deal of work has been completed over the past 20 years to incorporate consumer electronics into cutting-edge information technologies like the Internet of Things and wearable/implantable electronics.^1^ The most recent developments include on-tissue sensors, implantable and ingestible medical devices, environmental monitoring, as well as disposable plastic electronics such as smart food packaging, RFID tags, plastic cards, e-tickets, and many more.^2–9^ All these devices require abundant and inexpensive biofriendly materials and fabrication technologies supporting their sustainable development. Designing new materials with required functional properties has always been a topical issue in the materials science field.^10,11^ However, with a few notable exceptions,^12–14^ the vast majority of synthetic organic materials investigated for their function in electrical gadgets exhibit limited performance and stability in time and under operation conditions, and moreover only a few of them are safe for human use.^15–20^ Efforts of the scientific community include both development of useful design rules and establishment of reproducible fabrication of new materials, which is particularly important for the active component layers of electronics, *i.e.*, dielectrics, semiconductors, and encapsulates.^21,22^ While proof of principle studies have shown a great potential of organic materials in obtaining good performance in optoelectronic devices, combining various functional features in a single material (*i.e.*, low cost, large availability, non-toxicity, ease of processability, *etc.*) remains challenging.^23,24^ In particular, a lot of effort is presently invested into development of efficient and stable “green” materials for bioelectronics applications.^17,25–33^ In this work, we demonstrate that natural, commercially available waxes from plant origin (rice bran and carnauba) and animal origin (beeswax, lanolin and shellac) are viable, low-cost dielectric materials to be considered for the fabrication of low-operating voltage organic field effect transistors.

Beeswax (referred in short as BW in this study), scientifically known as *cera alba*, is a naturally occurring wax that is produced by worker bees. The hive workers manufacture the wax through their abdomen wax glands and use it as building material to produce cell walls where larvae are grown and honey is deposited. Beeswax is in fact an edible compound, with negligible toxicity, approved as food ingredient in many countries, while the European Union listed it as food ingredient with the E-number 901. Beeswax is a wax with a low melting point reported in the literature in the range of 62–64 °C. Constituently, the beeswax is composed mainly of long chain (C30–C32) esters of fatty acids as primary component (*i.e.*, palmitate, palmitoleate, and oleate esters) and various long-chain aliphatic alcohols as minor component (*i.e.*, triacontanyl palmitate and cerotic acid).^34^ The main application of beeswax was represented over the centuries by candle manufacturing, since it burns cleaner, brighter and slower than other waxes. Nowadays beeswax has found applicability in many areas, including medicine as surgical bone wax, in shoes, surfboards and furniture polishing as component material for various hydrophobic formulations.^35^

Carnauba wax (referred in short as CW in this study), known also as palm tree wax is a wax extracted from the leaves of the tree *Copernicia prunifera*, native to the northeast of Brazil. It consists mainly of aliphatic esters (∼40 wt%), diesters of 4-hydroxycinnamic acid (∼ 21.0 wt%), ω-hydroxycarboxylic acids (∼13.0 wt%), and fatty alcohols (∼12 wt%) with the chains in the range of 26 to 30 carbon atoms^36^. Carnauba wax is a wax of minimal toxicity that is considered safe when coming in contact with human skin or being ingested.^37^ In cosmetics, carnauba wax found various applications as a thickener for deodorants, lipsticks, eyeliners, eyeshadows, mascaras, and even sun protection creme applications. Due to its high melting point of ∼82–86 °C, carnauba wax found a plethora of applications as glossy finish for automobiles, shoes, dental floss, tobacco pipes, paper, musical instruments, floor planks and furniture to name only a few from a very exhaustive list. For the latter application as food gloss agent, carnauba wax received the E number 903 in the European Union.

Lanolin (wool wax),^38^ referred in short as LW in this study, is extracted from the wool of various domestic sheep breeds that are raised for wool production. Although many publications reporting medical drugs consider lanolin as wool fat (adeps lanae), in reality lanolin is not a true fat since it consists mainly of sterol ester and lacks glycerol esters.^39,40^ The composition of lanolin is rather uniform, consisting mainly of long chain waxy esters that account to approximately 97% of its weight, followed by alcohols, lanolin acids and lanolin hydrocarbons as minor constituents. With a melting point ranging between 38 °C to 44 °C,^41^ lanolin has a physical appearance of grease (margarine), and can be easily spread across surfaces with the aid of a solid object. In a similar role that it has in nature to protect the sheep wool and skin from the environment conditions, lanolin and synthetic derivatives of it found various applications in cosmetics for human skin protection and treatment of various medical conditions.^42^

Rice bran wax (referred in short as RBW in this study) is a by-product of the refining process of rice bran oil. The composition of rice bran wax is made of C46–C62 esters, C20–C36 fatty alcohols and C20–C26 fatty acids^43^. The aliphatic acids consist mainly of crystalline palmitates, and other lignoceric acids, followed by additional higher carbon content wax acids in various amounts. The higher alcohol esters comprise mainly ceryl alcohol and melissyl alcohol, both being high carbon content alcohol esters. In addition, rice bran wax contains squalene and phospholipids in minor contribution to its final composition. One of the main advantages of rice bran wax compared to synthetic waxes and many other animal waxes is its completely non-toxic character, rice bran wax being in fact edible. The melting point of rice bran wax is 75–86 °C, and can be used as a cheaper substitute for carnauba wax, a wax with similarly high melting point. In addition, rice bran wax is freely miscible with many other natural waxes. Rice bran wax found a long list of industrial applications in various industries, like for example cosmetics, food,^44^ pharmaceutical textile, *etc.*, mainly due to its miscibility and ease of formulations advantage.^45^ In electronics applications, the rice bran wax was recently reported as tablet lubricant.^46^

Shellac wax is a natural byproduct obtained during the processing of shellac resin. The insects of the species *Kerria lacca* produce the shellac secretion called in its raw form sticklac (*i.e.*, the crude form of lac resin before processing steps that transform it into a semi-refined product called seedlac). The shellac secretion produced by the insects is meant to protect them and the larvae from outside rain, moisture, sun, and UV radiation, and the encrustation itself is similar to honey or silk. Shellac can be considered a vegetarian product as no insects are harmed or killed during the collection process. The wax content in the sticklac secretion is typically 5–6% by weight. Since the primary focus of lac processing is shellac resin extraction and its subsequent purification, the wax is removed as a byproduct rather than being specifically targeted for extraction. The exact percentage of wax can vary depending on lac strain (*e.g.*, the host tree Kusmi lac *vs.* Rangini lac for example), collection method and processing conditions. When seedlac is used as the starting material, it can be refined by either physical bleaching (*i.e.*, solvent extraction process) or chemical bleaching. The refining process involves removal of the impurities and the waxes present in the seedlac. In the solvent extraction process, the seedlac is dissolved in a solvent and the wax is filtered out from the solution. The colour of the wax would depend on the colour/quality of the seedlac used. A decolorization process may or may not be used prior to filtration depending on the colour of the wax required. In the chemical bleaching process, the seedlac is dissolved in an aqueous medium and colour reduction is performed with the aid of various agents like sodium hypochlorite. Similar to the solvent extraction process, the filtration and removal of wax can be done either before or after the bleaching process. The colour of the wax would depend on the starting raw material and the bleaching process. Shellac wax has a slightly higher melting point than shellac resin and is considered to be a high melting point wax, together with carnauba wax and rice bran wax. It found diverse applications in cosmetics, food and pharmaceutical industries. Shellac wax is especially valued in cosmetics applications due to its smoothness, non-toxicity, and water resistance. It is used in lipsticks and balms as a binding agent. It is also used in hair styling products, nail polishes and varnishes. Shellac wax also finds application in car waxes, wood & leather polishes as well as lubricant and releasing agents. Although no electronic applications reports have been released so far for the shellac wax, the resin itself has a long history in science, especially as dielectric and substrate for the fabrication of organic field effect transistors.^47–52^ In this study we employed two shellac waxes, that differ from one another through their production process: the shellac wax solvent extracted (referred here in short as SWE) and shellac wax bleached (referred here in short as SWB). Although other waxes, like for example soybean, myrtle and candelilla were employed as substrates and encapsulates for electronics fabrication,^53^ the waxes employed in this work were not considered before as stand-alone dielectric materials for the fabrication of organic field effect transistors. In addition, despite the fact that parent shellac resin (highly soluble in ethanol) was reported before in many studies in the electronics field,^47–51^ and has a great potential for many industrial-relevant applications,^52^ the wax component of shellac is a novel material never reported for electronics fabrication studies.

## 2. Experimental

### Thermogravimetry (TGA) analysis

Thermogravimetric analyses of the analyzed waxes have been carried out with a TGA/PerkinElmer Q5000. The samples were weighted in platinum pans (∼5–27 mg, depending on the sample) and the measurements were performed in the thermal range from 70 °C to 900 °C, with a heating rate of 10 °C min^−1^ under nitrogen atmosphere (25 mL min^−1^). All the waxes have been measured using an identical experimental setup and procedure.

### Differential scanning calorimetry (DSC) analysis

Differential scanning calorimetry (DSC) was performed using a temperature-modulated calorimeter (DSC Q2000, TA Instruments, New Castle, DE, USA) equipped with an RCS90 cooling accessory. All the experiments were performed in hermetically sealed Tzero™ (TA Instruments, Lukens, DE, USA) aluminum pans under a dynamic nitrogen atmosphere. Temperature-modulated DSC was used to investigate the phase transitions of the analyzed waxes as follows: approximately 5 μg of a sample was first equilibrated at 200 °C and kept isothermally at this temperature for 5 min. The sample was then cooled down to 10 °C at a cooling rate of 2 °C min^−1^ and a temperature modulation of ±0.32 °C every 60 s. After another equilibration step (5 min at 10 °C), the sample was heated to 100 °C again with a heating rate of 2 °C min^−1^ and the same temperature modulation as before (second heating step). The evaluation of the thermograms was performed by the TA Universal Analysis 2000 software (TA Instruments, Lukens, DE, USA).

### Gel permeation chromatography (GPC) analysis

All the investigations of polymer properties were measured *via* GPC (Agilent 1100, Santa Clara, CA, USA) in chloroform (CHCl_3_). The analysis parameters were as follows: mobile phase flow 1 mL min^−1^; column temperature 23 °C, used column: PLgel 5 μm MIXED-C (300 × 7.5 mm). Measured polymer parameters were as follows: number-average molecular weight (*M*_N_), [g mol^−1^]; weight-average molecular weight (*M*_W_), [g mol^−1^]; dispersity.

### Elemental analysis

Elemental analyses for the waxes were performed on an Elementar Vario Micro Cube analyzer. Oxygen content was calculated for all samples by the difference in carbon, hydrogen, sulfur and nitrogen content. All determinations were done in duplicate. The standard deviation was always lower than 0.2 weight%. The analyses were carried out on 5 mg samples. The dynamic detection ranges of work in CHNS, CNS mode were:C 0.087–7 mg (absolute)H 0.010–1 mg (absolute)N 0.034–10 mg (absolute)S 0.039–2 mg (absolute)

The precision is less than/equal to 0.1% of the absolute values. The instrument was periodically calibrated for each operating mode, for each measured element, over the entire measuring range, by performing a simultaneous determination in CHN or CHNS mode of 2 mg of acetanilide or sulfanilamide (analytical standards). Furthermore, for each analysis session, the “daily factor” was determined on standard samples (acetanilide or sulfanilamide) in order to correct the calibration based on the atmospheric conditions (pressure, temperature) at the time of analysis.

### Fourier transform infrared (FTIR) spectroscopy

Fourier transform infrared (FTIR) spectra of the waxes were measured with iS50 FTIR spectrometer (Thermo Scientific, Waltham, MA, USA) at ambient temperature (in an air-conditioned room) with the built-in single-reflection diamond attenuated total reflectance (ATR) crystal. Each ATR spectrum was collected in the standard mid-infrared spectral region (4000–400 cm^−1^) as an average of 32 scans with a resolution of 4 cm^−1^ (data spacing 0.5 cm^−1^).

### ^31^P-NMR

Phosphitylation of samples was performed adapting the method described by Crestini *et al.*^54^ Samples were dried overnight in an oven set at 50 °C and then transferred in a desiccator until they reached room temperature. A mixture of pyridine and CDCl_3_ (1.6 : 1 v : v ratio) was prepared and dried over molecular sieves (3 Å). Using this mixture, a 0.1 M solution of the relaxation reagent, chromium(iii) acetylacetonate (5 mg ml^−1^), and of internal standard, cholesterol (40 mg ml^−1^), was prepared. All solutions were stored in the dark. About forty milligrams of sample were dissolved in 0.5 ml of solvent solution in a vial equipped with a stirring bar. Then, 0.1 ml of the internal standard and relaxation solution was added, and the solution was stirred for ∼12 hours to achieve the greater solubilization possible of the sample. At least, 28 and 32 mg were solubilized for SWB and SWE, respectively. Then, 0.1 ml of 2-chloro-4,4,5,5-tetramethyl-1,3,2-dioxaphospholane (TMDP) was added and the solution was kept under vigorous magnetic stirring for 30 minutes. The resulting solution was transferred into an NMR tube. ^31^P-NMR spectra were recorded on a JEOL YH spectrometer with a probe operating at 202.468 MHz at 25 °C in CDCl_3_. Chemical shifts were calibrated from the ^31^P NMR signal at 132.2 ppm arising from the reaction product between residual water and TMDP. Spectra were quantitative, and proton broadband decoupling was applied during the acquisition time. Cholesterol was used as an internal standard. Spectra were acquired with 100 ppm spectral width, 32 000 data points, 11 s relaxation delay, and 512 scans. The spectra were analysed using JEOL Delta software.

### Atomic force microscopy (AFM) and amplitude modulated kelvin probe force microscope (AM-KPFM) characterization

Atomic force microscopy (AFM) and amplitude modulated kelvin probe force microscopy (AM-KPFM) measurements were conducted on waxes films deposited on aluminum-coated glass substrates, utilizing an Asylum Research MFP-3D AFM system. For the AM-KPFM measurements, ASYELEC-01-R2 probes (Ti/Ir coating on both the reflective and tip sides) were employed, with a spring constant of 2.8 N m^−1^, resonant frequency of 75 kHz, and a tip radius of 25 ± 10 nm. Heights and contact potential differences (CPD) were obtained using a two-pass mode in the AM-KPFM setup, with natural wax films grounded during scanning (scan speed of 5 μm s^−1^, with the probe elevated by 10 nm during the second pass). Root mean square (RMS) values for both topographical roughness and CPD variations were calculated as averages with standard deviations, considering five arbitrarily selected 20 × 20 μm^2^ areas from each sample. Topographical and CPD images were analyzed using the open-source software Gwyddion v2.62.^55^ First-order line filtering was applied to topography images, along with base plane leveling, while only zero-order line filtering was used for CPD images.

Contact angle measurements with ultrapure water droplet were performed with a KRÜSS DSA 100 Contact-Angle Measuring System. In addition to the contact angle value, the measurement equipment was able to offer information regarding the film's surface energy. The liquid used were ultrapure water (a very polar liquid with a total surface tension *γ* = 72.8 mN m^−1^ separable in a polar component *γ*^P^ = 51 mN m^−1^ and a dispersive component *γ*^D^ = 21.8 mN m^−1^). Here are reported the mean values from five measurements with water for each wax.

### Waxes processing in thin films and OFETs fabrication

Beeswax was purchased from Aldrich, product no. 243248 and used in this work without further purification. Carnauba wax (product name Carnauba wax no. 1 yellow) was purchased from Aldrich, product no. 243213 and used also without further purification. Lanolin wax (product name Lanolin) was purchased from Sigma, product no. L7387 and used without further purification. Rice bran wax (product code LICOCARE RBW 102 FL) was obtained from Clariant Plastics & Coatings (Germany) GmbH. Shellac wax bleached and shellac wax by solvent extraction process were both purchased from AF Suter & Co. Ltd, and used without further purification. Both materials carry the production date of April 2019. All waxes were solubilized in chloroform; however, the bleached shellac wax had a very limited solubility in chloroform and was subsequently processed from *n*-octane for this study. All waxes were dissolved in their processing solvent in a concentration of 1 mg ml^−1^ and subsequently filtered through a hydrophobic membrane before their deposition as thin films. For the deposition process, we investigated drop casting, spin coating, and blade coating (also referred elsewhere as doctor blading) methods. However, only the latter method produced films of acceptable quality, since the evaporation of the solvent and the subsequent nucleation of the waxes were uncontrollable *via* drop casting and spin coating methods. For blade coating, a COATMASTER 509 MC device was used. The optimized parameters for operation of the blade coating device were a wax concentration in the carrier solvent of 1 mg ml^−1^, a blade height of 0.6 mm, a processing speed of 2.5 mm s^−1^ and an injection volume of 25 μl of precursor material on the back of the moving blade.

For the impedance measurements, the waxes were processed on top of 50 nm thick aluminum electrodes on glass substrates, and the metal–insulator–metal structure was terminated by the deposition of 50 nm top aluminum electrodes. Both aluminum electrodes were deposited *via* physical vapor deposition on an Edwards AUTO 306 Vacuum Coater at a deposition speed of 1 Å s^−1^.

The OFET structure employed in this work consists of a staggered bottom gate-top contact architecture, on glass substrates. The OFET schematic will be presented in this work. Each glass slide contains 4 individual transistors that have all in common a 50 nm thick aluminum gate electrode and the doctor bladed wax. The two semiconductors employed, *i.e.*, fullerene, C_60_ as n-type semiconductor and pentacene as p-type semiconductors were deposited on individual patches, each of 60 nm in thickness on top of the blade-coated wax, using a specialized organic evaporator, *i.e.*, a Vaksis Research and Development Evaporator. The semiconductor evaporation was performed at a rate of ∼0.1 Å s^−1^ in a vacuum of 1 × 10^−6^ mbar or lower. The structure was terminated with a pair of top electrodes, *i.e.*, aluminum for fullerene (C_60_), and gold for pentacene, respectively, in an Edwards AUTO 306 Vacuum Coater at a deposition speed of ∼0.1–0.2 Å s^−1^ for the first 5 nm of the deposited layer and ∼1 Å s^−1^ for the remaining 55 nm of the total thickness of 60 nm. Each individual patch of organic semiconductor had assigned an individual pair of source and drain (S–D) electrodes, as illustrated in the appropriate figure schematic. The OFET dimensions were: width (*W* = 2 mm), being in fact the width of the gate electrode, and length (*L* = 25 μm), the distance between the source and drain electrodes. The OFETs measurement was performed on an Agilent Technologies A1500B Semiconductor Device Analyzer probe station situated in a glove box, under nitrogen atmosphere.

## 3. Results and discussion

The list of waxes employed in this work is presented in Table 1. As can be seen in the table, all waxes were uniformly processed from chloroform solutions, with the only exception of shellac wax produced *via* the bleaching method, which was virtually insoluble in the respective solvent. The SWB was instead processed from *n*-octane with the temperature of the blade coating surface set at 85 °C. The other five waxes were processed from chloroform at room temperature, without heating the blade coating surface. Although being unsuitable for vacuum sublimation as a method of fabrication, and being in general difficult to process in thin films from their precursor solutions, we show in this work that these natural materials have practical applicability for electronics fabrication.

**Table 1:** Waxes and their processability conditions by blade coating method

Sample	Label	Processing solvent (1 mg ml^−1^)	Processing temperature
Beeswax	BW	Chloroform	Room temperature
Carnauba wax	CW	Chloroform	Room temperature
Lanolin wax	LW	Chloroform	Room temperature
Rice bran wax	RBW	Chloroform	Room temperature
Shellac wax (solvent extracted)	SWE	Chloroform	Room temperature
Shellac wax (bleached)	SWB	*n*-Octane	85 °C

**Fig. 1:**
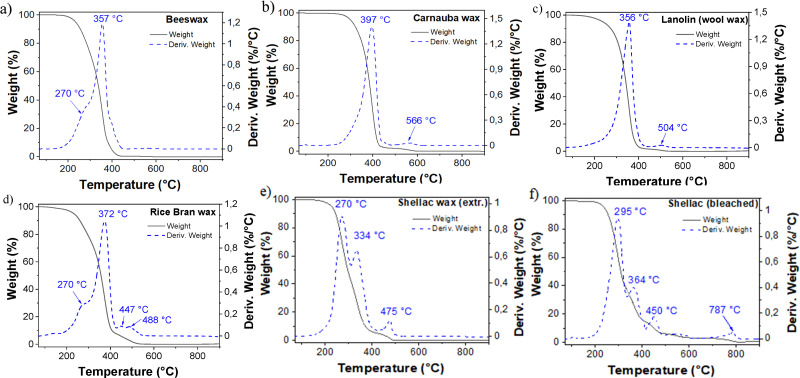
TGA analysis of the analyzed waxes in the range of 70 °C to 900 °C, under nitrogen atmosphere, with a uniform heating rate of 10 °C min^−1^. (a) Beeswax; (b) varnauba wax; (c) lanolin wax; (d) rice bran wax; (e) shellac wax solvent extracted; (f) shellac wax bleached.

Two methods of thermal analysis were utilized to reveal the physical structure and phase behavior of the studied natural waxes. In the first one, thermal stability of the analyzed waxes was determined by employing the thermogravimetric analysis. The thermograms of all six waxes revealed exothermic reactions that started occurring when the samples were heated above 100 °C under a nitrogen atmosphere (see Fig. 1). For the beeswax, displayed in Fig. 1(a), a negligible first weight loss was detected below 100 °C (∼0.01%) corresponding to possible adsorbed water traces. This wax started losing weight around 150 °C, but the beeswax was not completely decomposed until the temperature reached 430 °C. The most pronounced weight loss for beeswax began at temperatures around 200 °C, centered at 357 °C, where the sample lost almost all its weight (∼99.7%), and the process continued with minimal additional loss until 450 °C. In this temperature range, another decomposition temperature could be detected at 270 °C. From 450 °C to the end of the measurement (*i.e.*, until 900 °C), a negligible residual weight was detected due to a complete decomposition of the wax. The beeswax degradation under nitrogen evaluated in this work shows similar thermal decomposition pattern with the material already reported in the literature.^56^ Carnauba wax (shown in Fig. 1(b)), had a first weight loss detected below 100 °C (<0.1%), in line with all the other waxes, due to adsorbed moisture. The second important weight loss is centered at 397 °C, where the sample lost ∼97.1% of its weight, finishing at 450 °C, in agreement with the data reported in the literature for this particular wax.^57,58^ In this work, a second decomposition temperature was detected at 566 °C, with a weight loss of ∼2.7%, due to a complete decomposition of the wax until slightly above 600 °C. Remarkably, this wax showed the highest decomposition temperature when compared with the other samples investigated.

Lanolin wax (Fig. 1(c)), showed very similar thermal decomposition behavior to carnauba wax (see Fig. 1(b)). Here, again a slight weight loss most likely due to adsorbed atmospheric moisture was detected below 100 °C (<0.1%). The second and more important weight loss is centered at 356 °C, where the sample lost ∼98.4% of its weight until 450 °C. As in previous analyzed waxes, a small weight loss of ∼1.6% was detected at higher decomposition temperatures between 450 °C and 600 °C, *i.e.*, centered at 504 °C, followed by a similar final weight loss after 650 °C. The remnant weight of this wax is ∼0.4%. As for carnauba wax, a thermal decomposition within a narrow temperature range proves the purity of the wax. Rice bran wax (Fig. 1(d)) had also a first weight loss detected below 100 °C (<0.1%), from possible traces of adsorbed water. The second main weight loss was centered at 372 °C, however, at 270 °C a further decomposition temperature was detected. The sample lost approximately ∼92% of its weight up to 400 °C. Slightly different from previously mentioned waxes, two further decomposition temperatures were detected at 447 and 488 °C, corresponding to a combined weight was lost less than 8%. From 600 °C to the final measurement temperature of 900 °C, only a residual weight of ∼0.3% was detected, attributed to a nearly full decomposition of the sample, similarly to all the other measured waxes. Two variations of Shellac wax (solvent extracted and bleached), shown in the thermogram of Fig. 1(e) and (f), displayed also a very small weight loss below 100 °C (<0.05%) due to the release of adsorbed moisture. Similar to one another, both materials presented three decomposition temperatures from 200 to 600 °C. Chemically, Shellac wax contains a number of carboxylic acid and hydroxyl groups,^59^ and its degradation by temperature increase occurs in three steps, with a complete degradation taking place when reaching a temperature of *ca.* 600 °C, in agreement with published reports.^60^ It was observed that the main weight loss was centered at 295 °C and 270 °C, for bleached and solvent extracted samples respectively. This first loss event is followed by two higher decomposition temperatures (*i.e.*, 364 °C and 334 °C, as well as 450 °C and 475 °C, for bleached and extracted samples, respectively). In this temperature range, the shellac samples lost their main weights, corresponding to *ca.* 90% for the bleached and 96% for the solvent extracted sample. Important to mention is that the bleached shellac wax sample presented a last decomposition temperature at 787 °C, with a weight loss of 2.7%, possibly due to traces from the chemical compound retained by the material during the sample production step. A summary of the TGA analysis is presented in Table 2. One can see a similar decomposition process under heating for all the waxes that starts at 200 °C. Noticeably, the lowest decomposition temperature and thus, the materials with lowest thermal stability were the two Shellac waxes (at 270 °C and 295 °C for extracted, SWE and bleached, SWB, respectively), in agreement to reported studies.^61^ SWE and SWB are different than the other four waxes on their TGA behavior, since in the latter group only one main decomposition peak was detected within a narrower temperature range, which may be indicative of their high purity. The highest degradation temperature of the 6 waxes occurs at a weight loss maximum close to 400 °C in the case of carnauba wax, demonstrating that this wax has the best thermal stability performance among the others investigated in this pool, a fact to be expected.

**Table 2:** Comparison of the waxes with respect to the higher decomposition temperature, *T*_d_, and their corresponding weight losses between two temperature ranges, from 100 to 600 °C

Waxes	*T*_d_/°C	Weight loss/%	Weight loss/%
		(100–450 °C)	(450–600 °C)
Beeswax (BW)	357	99.64	0.46
Carnauba wax (CW)	397	97.13	2.71
Lanolin (wool wax) (LW)	356	98.35	1.62
Rice bran wax (RBW)	372	92.27	7.69
Shellac wax (extracted) (SWE)	270	96.40	3.86
Shellac wax (bleached) (SWB)	295	89.99	6.78

**Fig. 2:**
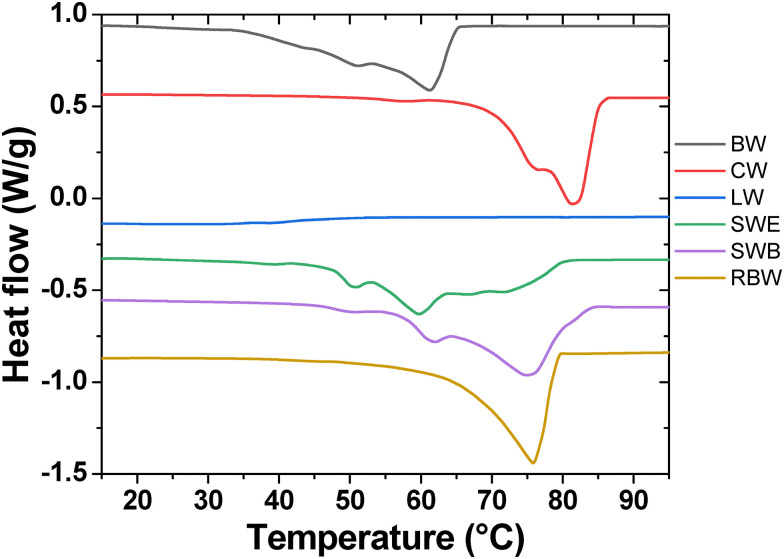
DSC thermograms from the second heating step for all tested waxes. Curves are offset for clarity.

The second method of thermal analysis employed to reveal the physical structure and phase behavior of the studied natural waxes is differential scanning calorimetry (DSC). DSC thermograms, presented in Fig. 2, confirm that all waxes except for LW show an obvious phase transition endotherm in the analyzed temperature range. Apparently, LW components do not form any crystalline structures in the solid state. While in the case of the RBW a single endotherm was detected, for BW, CW, SWE, and SWB a complex phase transition behavior of the waxes is demonstrated by the complex overlapping endotherm signals. The first phase transition represented by the weak endotherm signal at the lowest temperature range (30 –50 °C) is commonly assigned to a change of the crystalline structure (*i.e.*, s–s transition) of paraffin content of a wax.^59^ Solid–liquid (*i.e.*, melting) transitions of the individual wax components occur at the higher temperatures in the order of a strength of their cohesion forces (paraffins → esters → fatty alcohols → fatty acids). Among the analyzed waxes, the lowest thermal stability (as represented by the onset of melting) was confirmed for the BW while the highest melting onset was detected for CW. From the comparison of the melting signals of samples SWE and SWB, it is evident that the bleaching procedure increases the solid-state thermal stability of the respective shellac wax, most likely *via* an increase in the relative content of more thermostable constituents (free fatty acids and fatty acid salts), as indicated already by the TGA study and by the FTIR results that will follow. The wax melting point is usually assigned to the peak temperature of the whole melting signal. Although the use of a single melting temperature is not very suitable for describing the complex melting behavior exhibited by waxes, for a basic comparison we can rank the analyzed waxes in order of increasing melting point as follows: SWE (59 °C) < BW (61 °C) < SWB ≈ RBW (75 °C) < CW (81 °C). The highest melting point of CW may be explained by the highest reported average carbon chain length (∼50 carbon atoms, compared to, *e.g.*, ∼40 carbon atoms for BW).^62^ The aromatic content of CW may also play a role in its thermal stability. Furthermore, the highest melting point of CW is also in good agreement with its highest thermochemical stability represented by the decomposition temperature *T*_d_ (see the results of TGA, Fig. 1).

**Fig. 3:**
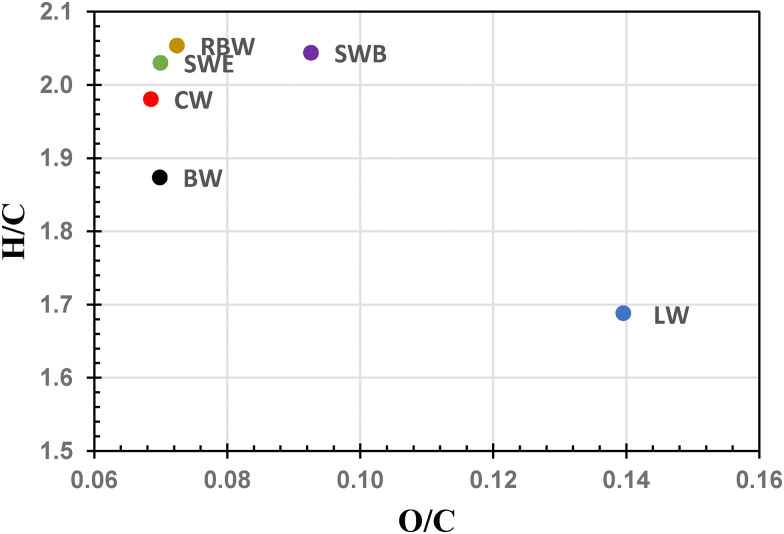
Van Krevelen plot of the results of elemental analysis of the natural waxes.

**Fig. 4:**
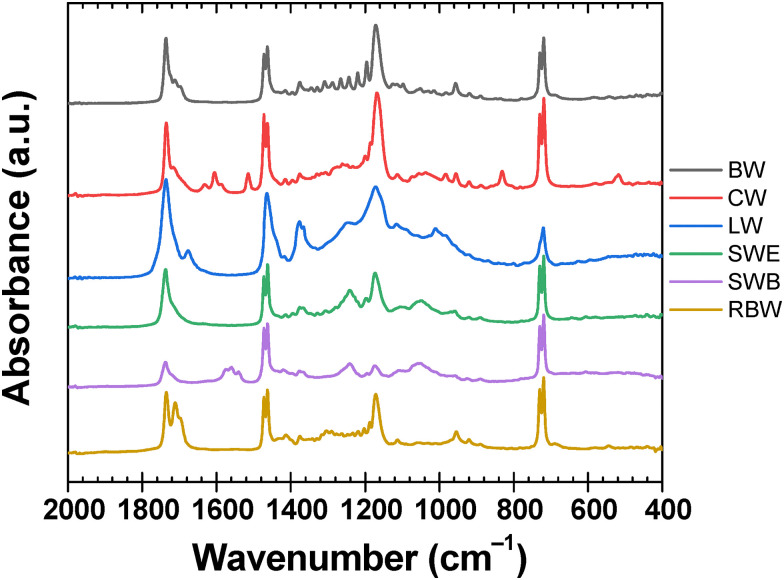
ATR-FTIR spectra of the analyzed waxes in the spectral range 2000–400 cm^−1^.

Combination of elemental analysis (see Table 3 and Fig. 3) and FTIR investigations provide valuable insights into chemical structure of the studied natural waxes. The ATR-FTIR spectra (see Fig. 4) confirm the highly heterogeneous composition of all waxes that combines the long-chain alcohols, saturated and/or unsaturated free fatty acids and their esters, and long-chain hydrocarbons. The ATR-FTIR spectra are in good agreement with other reported FTIR analyses of these natural materials.^63,64^ Commonly, the FTIR spectra are dominated by the intensive doublets at around 2900, 1470, and 720 cm^−1^, that correspond to stretching, bending, and rocking, respectively, of the –CH_2_– groups in long carbon chains. Other dominant spectral features are attributed to stretching of C=O in esters (1735 cm^−1^) and free fatty acids (1720–1700 cm^−1^), and also the C–O–C stretching at 1170 cm^−1^ that provides another spectral signature of esters. Despite basic spectral similarity of all materials, interesting structural differences among the studied waxes can be revealed from a more detailed interpretation of these spectra. Firstly, the relative intensities of the dominant carbonyl stretching band (1735 cm^−1^) and the neighboring shoulder peak at 1710 cm^−1^ indicate that the ratio of free acids-to-esters decreases among the studied waxes in the order RBW ≈ SWB > BW > CW ≈ SWE > LW. Relatively lower content of free fatty acids in LW and SWE should be considered with care. In the case of LW, the carbonyl stretching band is shifted to 1680 cm^−1^, which may be attributed to α,β-unsaturated acids. Similarly, the content of lower free fatty acids in SWE may be understood considering the signal at 1530–1580 cm^−1^ that represents the dissociated –COO^−^ groups in fatty acid salts. Nevertheless, the overall increase in either free or substituted carboxyls, brought by the procedure of bleaching to the structure of shellac wax, is indicated not only by FTIR but also by the corresponding increase in O/C molar ratio determined by elemental analysis (see Table 3 and Fig. 3). Regarding the other oxygen-containing functionalities, highest content of alcohols in LW is represented by the broad O–H and C–O stretching bands above 3000 cm^−1^ and 1000 cm^−1^, respectively. The high alcohol content provides the lanolin LW with the highest relative oxygen content among all studied waxes (see its highest O/C ratio in the Van Krevelen plot shown in Fig. 3). Aside from the oxygen-containing groups, the structural analysis provided interesting information also on the carbohydrate structural moieties. Firstly, most of the studied waxes exhibit the value of elemental ratio H/C close to 2, which confirms the methylene –CH_2_– group as the most prominent carbohydrate motif in their structure. In FTIR spectra, this is supported not only by the already mentioned strong FTIR absorption at 2900, 1470, and 720 cm^−1^, but also by the typical pattern of sharp absorption bands in 1400–1200 cm^−1^, that are assigned as C–C–C skeletal vibrational modes and wagging and twisting vibrations of methylenes. Lower H/C contents of BW and LW, together with their noticeable contents of sulphur and nitrogen indicate presence of other organic contaminants in these materials beyond the major wax constituents (paraffins and long-chain alcohols, fatty acids and their esters). In CW, some aromatic constituents signal their presence by the typical skeletal ring breathing modes at 1630–1600, 1510 and 830 cm^−1^. These aromatic components are most likely derivatives of cinnamic acids as repeatedly reported for carnauba waxes^65^.

**Table 3:** Elemental analysis of the natural waxes (results shown as atomic %)

	N (at%)	C (at%)	H (at%)	S (at%)	O_calc_ (at%)	H/C	O/C
BW	0.015	34.0	63.6	0.029	2.4	1.874	0.070
CW	0.000	32.8	64.9	0.006	2.2	1.980	0.069
LW	0.008	35.3	59.7	0.069	4.9	1.688	0.140
RBW	0.000	32.0	65.7	0.000	2.3	2.054	0.072
SWE	0.000	32.3	65.5	0.000	2.3	2.030	0.070
SWB	0.000	31.9	65.2	0.006	3.0	2.044	0.093

In parallel with the insights gained from elemental analysis and FTIR investigations described above, the gas permeation chromatography (GPC) analysis further elucidates the molecular weight distribution of these heterogeneous waxes. The results reveal that the natural waxes, comprised of complex mixtures of long-chain fatty acid esters, alcohols, and hydrocarbons, exhibit distinct number-average molecular weights (*M*_N_) spanned from 688 to 1 389 g mol^−1^; and weight-average molecular weights (*M*_W_) from 940 to 1 822 g mol^−1^, with dispersity values ranging from 1.151 to 1.790, indicative of moderate heterogeneity (see Table 4). Notably, differences in the GPC-derived parameters correlate with variations in chemical composition as revealed by the FTIR: for instance, the relative intensities of the carbonyl and methylene bands, as well as by the elemental O/C ratios. It is important to emphasize that, owing to the use of a polystyrene calibration standard, the absolute *M*_N_/*M*_W_ values obtained are inherently relative, serving best for comparative assessment among the waxes rather than as definitive molecular weight measurements. Rice bran wax (RBW) exhibits the highest *M*_N_ with the narrowest distribution, indicating a relatively uniform molecular composition. In contrast, Carnauba wax (CW) stands out with a notably broader molecular weight distribution, reflecting a more heterogeneous mixture of constituents. This comparative framework, when integrated with the complementary spectroscopic and elemental analyses, provides a robust understanding of the subtle molecular differences that underpin the diverse physical properties of these bio-based materials.

**Table 4:** Polymer properties of the waxes measured *via* GPC

Waxes	*M*_N_ [g mol^−1^]	*M*_W_ [g mol^−1^]	Dispersity [−]
Beeswax (BW)	1.097	1.436	1.309
Carnauba wax (CW)	1.018	1.822	1.790
Lanolin (LW)	811	1.180	1.455
Rice bran wax (RBW)	1.389	1.599	1.151
Shellac wax-solvent extracted (SWE)	827	1.207	1.459
Shellac wax-bleached (SWB)	688	940	1.367

**Fig. 5:**
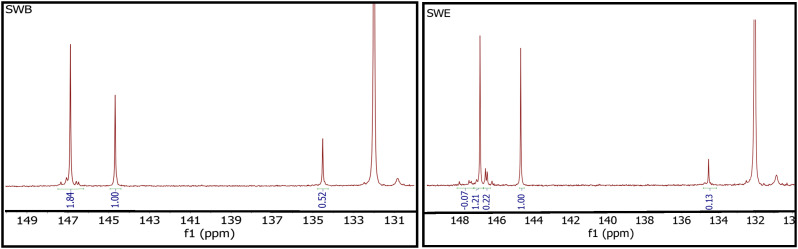
^31^P NMR spectra displaying the relevant signals in the spectral range 130–150 ppm for SWB (left panel) and SWE (right panel).

^31^P NMR spectra can offer a quantitative estimation of alcoholic and carboxylic groups on the molecular skeleton, after functionalization with a proper phosphitylation agent. However, this piece of information is reliable only if the starting material can be fully dissolved in the deuterated solvent (deuterated chloroform: pyridine employed in this study), and the only waxes soluble in the respective solvent in this study were the two shellac waxes. Therefore, the ^31^P NMR analysis was applied to SWB and SWE to confirm the nature of functional groups enriched (or discarded) during the extraction process. ^31^P NMR spectra contain the relevant signals in the spectral range 130–150 ppm (see Fig. 5). The calculated hydroxyl content is reported as mmol per gram of analysed sample in Table 5. Integration is referred to cholesterol at 144.8 ppm, used as an internal standard. As expected, the two shellac waxes present only aliphatic hydroxylic groups and carboxylic acids. In the case of SWB, the number of carboxylic acids is ∼25% of aliphatic hydroxyl groups, whilst in SWE it is lower. This demonstrates the lower solubility of free carboxylic acids in the extraction solvent used for the shellac wax fractionation. The greater contribution of aliphatic OH is related to secondary alcoholic groups in both samples. In the extracted shellac wax (SWE) the ratio between primary and secondary alcohols changes in favour of the secondary ones as the peaks observed ∼146.5 ppm become more evident.

**Table 5:** Calculated hydroxyl content for the two shellac waxes investigated in this study

Sample	Name	Aliphatic OH^a^	Phenolic OH^b^	Carboxylic acid^c^
1	SWB	0.69	0.00	0.18
2	SWE	0.50	0.00	0.06

a Integration limits: 149.0–145.0 ppm.

b Integration limits: phenolic hydroxyl content, 144.0–137.4 ppm.

c Integration limits: 136–133.6 ppm.

### Atomic force microscopy. Kelvin probe force microscopy

**Fig. 6:**
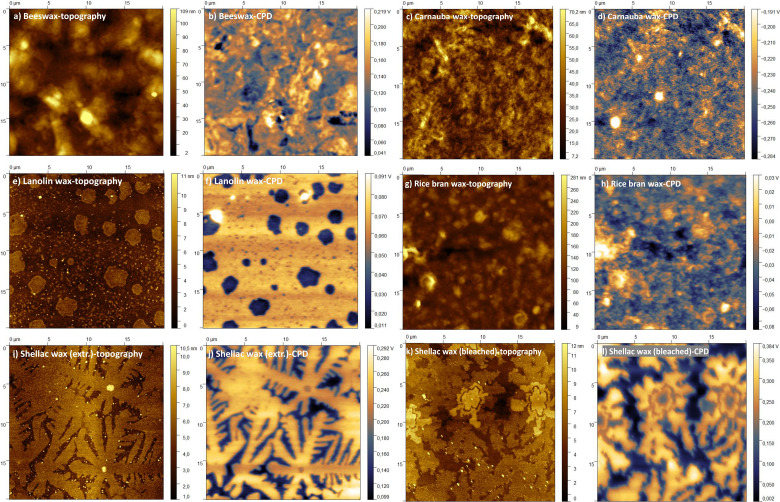
AFM-KPFM of investigated waxes: (a) beeswax topography; (b) beeswax contact potential difference; (c) barnauba wax topography; (d) barnauba wax contact potential difference; (e) lanolin wax topography; (f) lanolin wax contact potential difference; (g) rice bran wax topography; (h) rice bran wax contact potential difference; (i) shellac wax-solvent extracted topography; (j) shellac wax-solvent extracted contact potential difference; (k) shellac wax-bleached topography; shellac wax-bleached contact potential difference.

Fig. 6(a) and (b) show the typical topography and contact potential differences (CPD) of BW film. Contact potential difference substantiates the reactivity of the dielectric surface, *i.e.*, the charge trapping effect at the respective interface towards the semiconductor material. In the case of inorganic dielectrics, this trapping is more pronounced and results in the occurrence of hysteresis and lower recorded transistor current.^66^ Nevertheless, the CPD is important to know also for organic dielectrics, since it offers valuable pieces of information about the surface quality of the dielectric film, apart from its classic surface roughness (topography) investigation.^74,76^

**Fig. 7:**
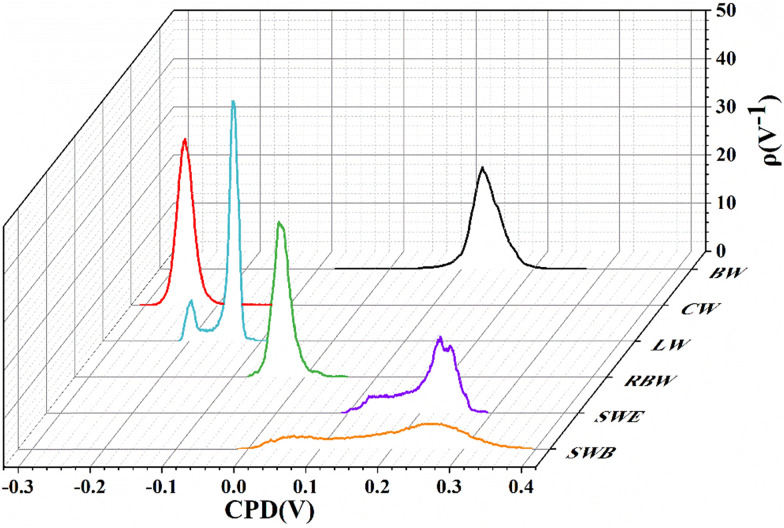
The distribution probability of CPD values for the analyzed waxes.

The surface of BW is composed of a large number of micrometer-sized sized “hills” and “valleys” with maximum height difference of ∼100 nm. The CPD image of BW shows no obvious correlation with the topography. The RMS roughness of topography averaged over 5 different surface areas is ∼19.8 ± 5.5 nm, while the CPD roughness is 30.5 ± 13.8 mV. The surface of CW is smoother than BW (the maximum height difference is ∼70 nm), the RMS height roughness is 9.6 ± 1.2 nm at five different surfaces, shown in one such example in Fig. 6(c). The CPD image of CW shows obvious positive correlation with the topography, with the RMS of CPD of 20.7 ± 6.0 mV, shown in Fig. 6(d). For LW, its surface is the flattest of all 6 wax samples investigated, as shown in Fig. 6(e), with the height roughness being ∼1.5 ± 0.2 nm. There are many small islands of different sizes (*i.e.*, from 100 nm to ∼2–4 μm) and heights less than 10 nm, randomly distributed on the surface. It is worth noting that the island shows lower surface potential, as seen in Fig. 6(f). By checking the distribution of the CPD (Fig. 7), the existence of two peaks confirms that there are two types of organic compounds present with different work functions. So, the CPD roughness is ∼16.1 ± 3.8 mV. As for RBW, shown in Fig. 6(g), it has the roughest surface of all 6 wax samples, the maximum height difference being greater than 200 nm, resulting in a RMS height roughness ∼32.9 ± 9.6 nm. The CPD image of RBW shows obvious correlation with the topography with the RMS of CPD of 12.7 ± 2.0 mV, displayed in Fig. 6(h). Fig. 6(i) shows big (*i.e.*, greater than 10 μm), snowflake-like hexagonal island structures, with a height of ∼6 nm for SWE. The SWE surface is very flat, the RMS of height roughness being ∼3.6 ± 2.1 nm, that makes the SWE as the third smoothest wax of this study with respect to topography. The CPD shows strong correlation with the topography as presented in Fig. 6(j). From the CPD distribution Fig. 7, at least three different peaks can be decomposed from the curve, resulting in the largest CPD roughness of ∼31.9 ± 9.2 mV. The SWB on the other hand, presented in Fig. 6(k) shows totally different topography compared to SWE. The island-like structure loses its hexagonal symmetry and instead forms an irregular flower-like structure, although the RMS of its height roughness is completely consistent with SWE, *i.e.*, ∼2.8 ± 1.8 nm, making the SWB being the second smooth wax of the study. From the CPD distribution of SWB, presented in Fig. 7, two peaks can be confirmed, attributed to two types of organic compounds, that result in a large CPD roughness of ∼67.8 ± 13.3 mV for SWB.

The *X* axis in Fig. 7 is related to the *Z* values of CPD image shown in Fig. 6 (panels b, d, f, h, j and l respectively) for the 6 waxes. For the clarity of the graph presented in Fig. 7 taking BW for example, its CPD ranges from −0.07 V to 0.27 V, which represent the minimum and maximum CPD values presented in Fig. 6(b) respectively. However, the CPD values in most areas are mainly distributed around 0.13 V, and these values represent the peak positions in Fig. 7 for BW. The values presented on the back axis as *ρ*, expressed in 1/*V* describe the distribution probability of CPD. The integrated area under each of the six curves is 1, and consequently, the values are expressed on the axis as 1/*V*. Similarly, the CPD roughness values presented in the Table 6 are mean values and their standard deviation comes from averaging over five measurements for each wax sample, since absolute CPD measurements are only in rare cases possible.^67^ Consequently, the CPD of the wax samples are all measured in different voltage ranges, and the corresponding ρ values displayed on the back axis of Fig. 7 are their distribution probability, the shapes of the six curves differs significantly from one another; it is either a sharp distribution (as in the cases of CW, LW, and RBW) or a broad distribution (as in the cases of BW, SWE, and SWB). For the sense of consistency, taking BW for example, the CPD roughness of BW is 30.5 ± 13.8 mV, where 30.5 is the mean value and 13.8 represents the standard deviation for the five surfaces measured on the BW sample.

**Table 6:** Height roughness (nm) and respectively the contact potential difference (CPD, expressed in mV) for the analyzed waxes

Sample	Height roughness (nm)	CPD roughness (mV)
BW	19.8 ± 5.5	30.5 ± 13.8
CW	9.6 ± 1.2	20.7 ± 6.0
LW	1.5 ± 0.2	16.1 ± 3.8
RBW	32.9 ± 9.6	12.7 ± 2.0
SWE	3.6 ± 2.1	31.9 ± 9.2
SWB	2.8 ± 1.8	67.8 ± 13.3

**Fig. 8:**
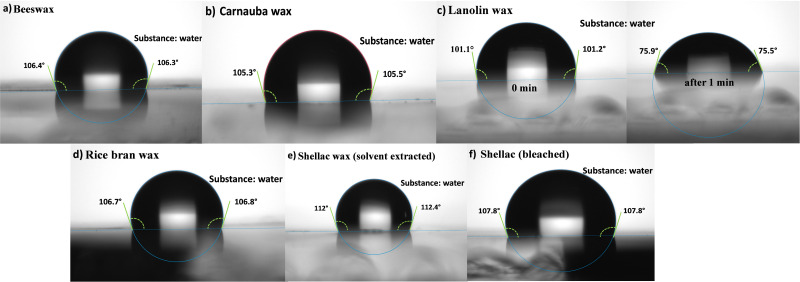
Contact angle of the investigated waxes with water droplet. (a) Beeswax; (b) carnauba wax; (c) lanolin wax; (d) rice bran wax; (e) shellac wax solvent extracted; (f) shellac wax bleached.

The contact angle with water droplet for the investigated waxes is displayed in Fig. 8. The waxes surfaces are definitely hydrophobic, the respective values of the contact angle being ∼106° for BW, ∼105° for CW, ∼107° for RBW, ∼112° for SWE and ∼108° for SWB. An interesting case was represented by LW, where the initial hydrophobic surface (*i.e.*, initial contact angle of ∼101° with water), turned into a hydrophilic one (*i.e.*, with a measured values of ∼75°) in no more than 1 minute time span, when the second droplet was deposited for the establishment of the statistical pool of data. Lanolin wax was in essence the only unstable wax when contacted to water.

**Fig. 9:**
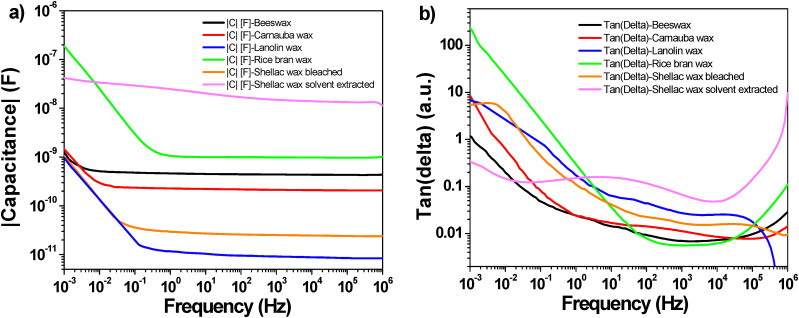
Impedance spectroscopy of waxes in MIM structures with aluminum top and bottom electrodes measured in a frequency window spanning from 1 MHz to 1 mHz: (a) modulus of capacitances; (b) loss angles.

Impedance spectroscopy helps understanding interface processes, particularly those involving modifications of the mechanical, electrical, compositional, or crystallographic characteristics of the system investigated. By examining their impact on the electrical conductivity of the system, it also aids in the comprehension of polarization and other changes in electrical characteristics.^68–70^ The benefit of impedance spectroscopy is that it allows for the measurement of conductivity across a range of frequencies, which makes possible learning about conductive species and pathways. These details are crucial for understanding the conduction mechanisms in analyzed materials. The impedance spectroscopy study of the waxes is presented in Fig. 9(a) that displays the capacitances of the waxes (in metal–insulator–metal, (MIM) structure) measured over a wide frequency range spanning over nine decades, from 1 MHz to 1 mHz, and in Fig. 9(b) that displays the associated loss angle (tangent delta) of the measurements. The split of the capacitances and loss angles in two separate graphs was preferred in order to improve the clarity of the figures.

The measured capacitances are in general uniform for all waxes, with a relatively flat plateau going down to ∼10 mHz applied frequency, followed by a steep increase, that was more prominent in the case of RBW. A similar observation can be obtained from inspecting the loss angle curves in Fig. 9(b), where again the RBW and SWB showed a relatively high loss angle value at the minimum measured frequency of 1 mHz. The SWB is in addition the only dielectric that clearly displays a relaxation (dome shape) in the low frequency range measurement of the loss angle, a fact that is indicative of the presence of ionic impurities in the material^71^. This event represents a drawback for this material, and this issue will be discussed further in the transistor measurement section. Because of the high dispersity of the thicknesses on the fabricated samples (an issue mentioned in detail on the transistors fabrication section), we could not determine accurately the breakdown field and the dielectric constant of the analyzed waxes.

**Fig. 10:**
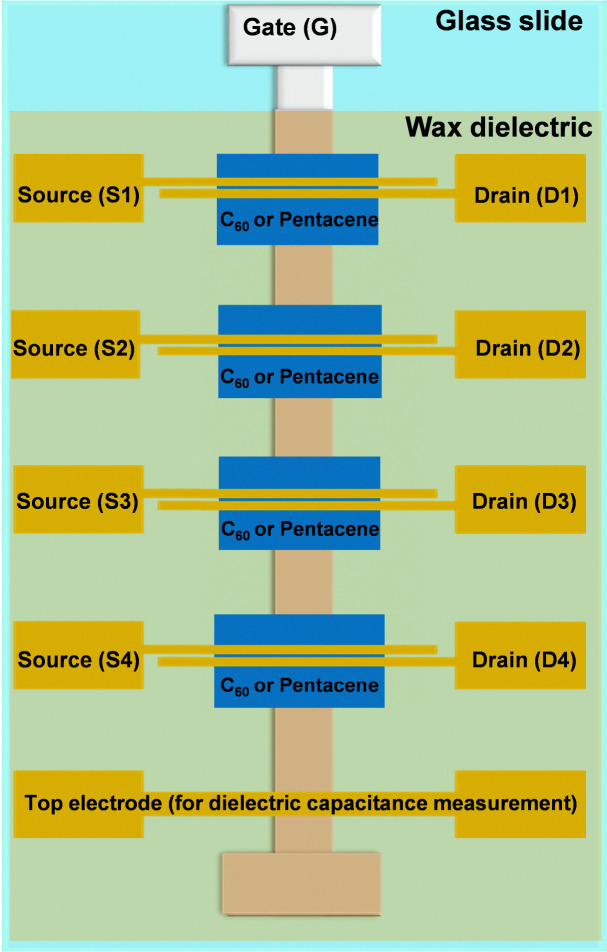
OFET schematic utilized in this work. We utilized a sequence of evaporations through 3 individual shadow masks: a gate mask that developed the gate electrode, a semiconductor mask that developed 4 individual patches of the organic semiconductor (*i.e.*, either pentacene or C_60_), and a source–drain mask for the development of 4 pairs of the top contacts and one continuous contact for the dielectric specific capacitance measurement. The final structure on one glass slide contains 4 individual OFETs, labeled accordingly in the schematic. The OFET dimensions were: width (*W* = 2 mm) and length (*L* = 25 μm).

The OFETs were fabricated on glass substrates, cut from the microscope slides (Paul Marinefeld GmbH & Co. KG) using a glass cutting bench. After scrupulous cleaning, they were dried and utilized for the deposition of gate electrodes (50 nm aluminum) in a physical vapor deposition system in a glove box (Edwards AUTO 306 Vacuum Coater) at a rate of ∼10–20 Å s^−1^. The fast evaporation rate for the gate electrodes is desirable over a slow evaporation rate, since its produces much smoother metal layers, a fact observed and reported previously by our group.^72,73^ The waxes were deposited from an appropriate solvent as described in the Experimental section, using a blade coating instrument. The two semiconductors investigated in this work (fullerene, C_60_, and pentacene) were deposited in a 60-nm-thick films at a rate of 0.1 Å s^−1^ in individual square patterns for each of the four OFETs on the glass slide (shown in Fig. 10), in a Vaksis Research and Development Engineering organic evaporator at a typical pressure of ∼ 1 × 10^−6^ mbar. Appropriate contact electrodes for source and drain (*i.e.*, gold for pentacene and aluminum for C_60_) terminated the device fabrication. The mask design contains also a continuous electrode (*i.e.*, a metal–insulator–metal structure, or MIM) necessary for the measurement of the capacitance of the dielectric, thought to be employed in the organic semiconductor field effect mobility calculation.

**Fig. 11:**
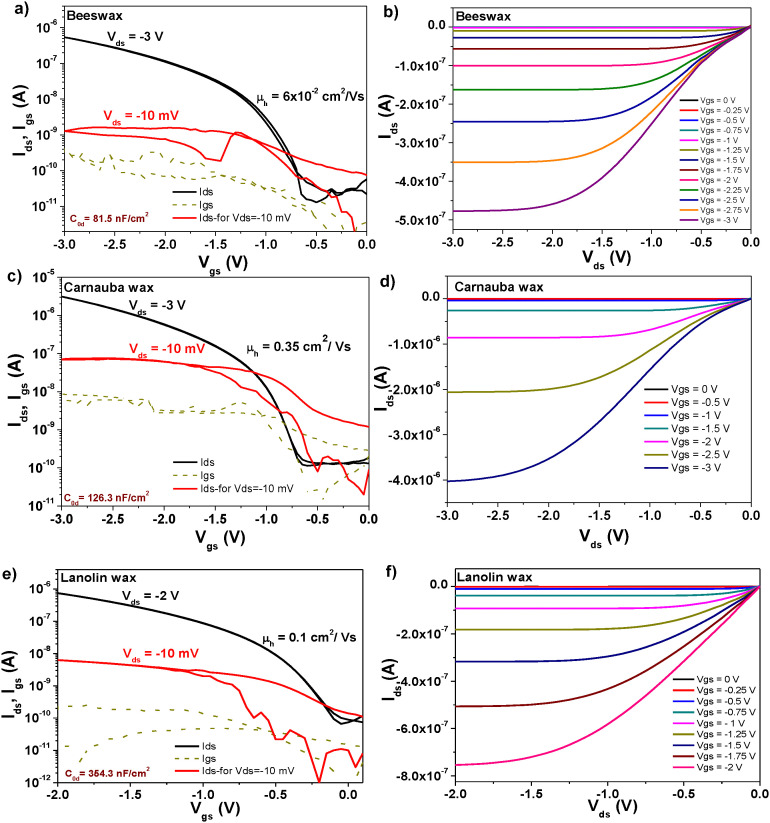
(a) Transfer and (b) output characteristics of an OFET with pentacene semiconductor and beeswax dielectric on plain aluminum gate electrode; (c) transfer and (d) output characteristics of an OFET with pentacene semiconductor and carnauba wax dielectric on plain aluminum gate electrode; (e) transfer and (f) output characteristics of an OFET with pentacene semiconductor and lanolin wax dielectric on plain aluminum gate electrode.

**Fig. 12:**
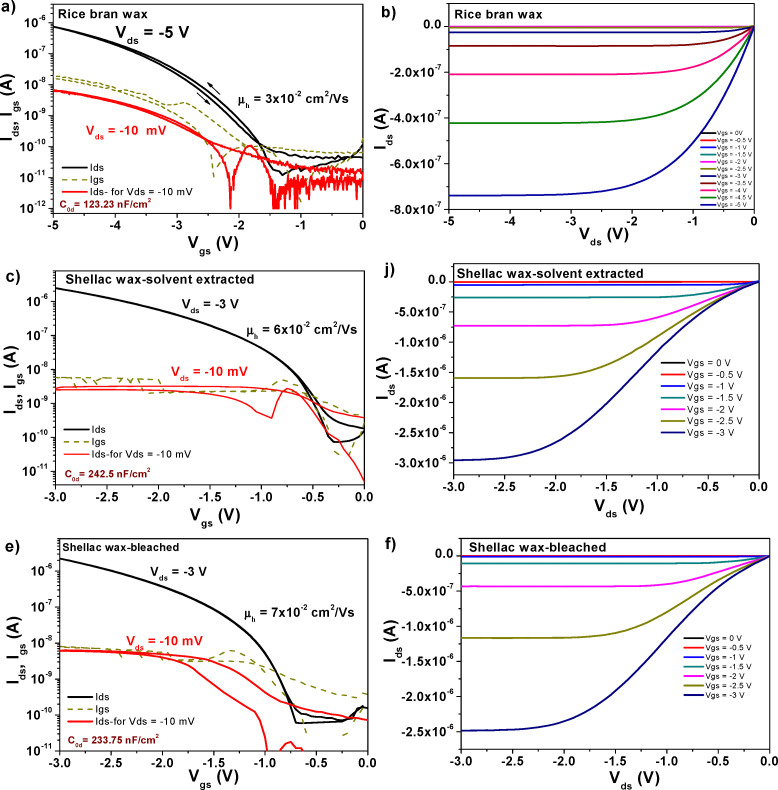
(a) Transfer and (b) output characteristics of an OFET with pentacene semiconductor and rice bran wax dielectric on plain aluminum gate electrode; (c) transfer and (d) output characteristics of an OFET with pentacene semiconductor and solvent extracted shellac wax dielectric on plain aluminum gate electrode; (g) transfer and (h) output characteristics of an OFET with pentacene semiconductor and beached shellac wax dielectric on plain aluminum gate electrode.

**Fig. 13:**
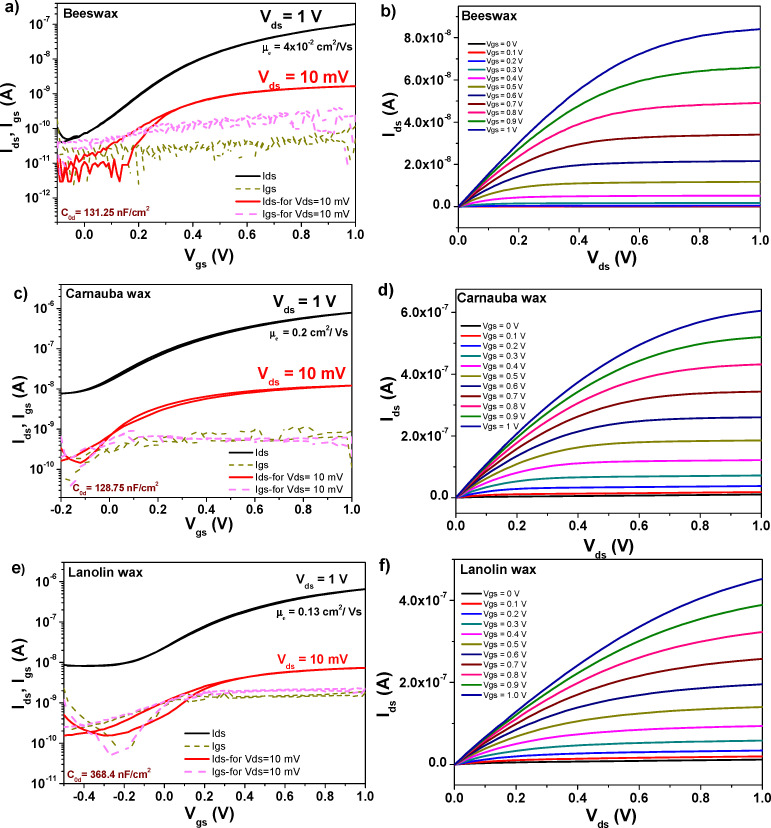
(a) Transfer and (b) output characteristics of an OFET with C_60_ semiconductor and beeswax dielectric on plain aluminum gate electrode; (c) transfer and (d) output characteristics of an OFET with C_60_ semiconductor and carnauba wax dielectric on plain aluminum gate electrode; (e) transfer and (f) output characteristics of an OFET with C_60_ semiconductor and lanolin wax dielectric on plain aluminum gate electrode.

**Fig. 14:**
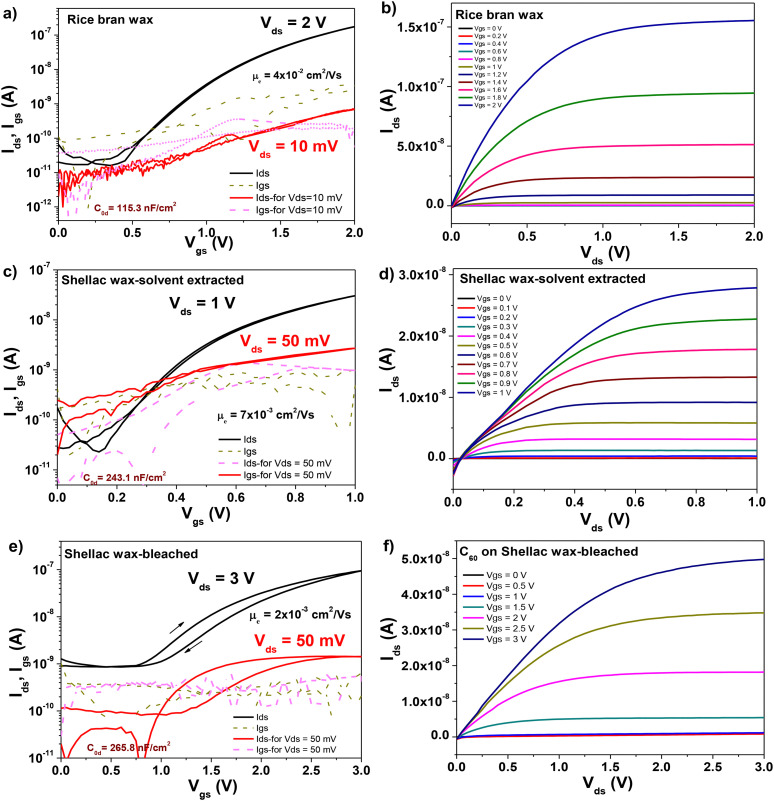
(a) Transfer and (b) output characteristics of an OFET with C_60_ semiconductor and rice bran wax dielectric on plain aluminum gate electrode; (c) transfer and (d) output characteristics of an OFET with C_60_ semiconductor and solvent extracted shellac wax dielectric on plain aluminum gate electrode; (e) transfer and (f) output characteristics of an OFET with C_60_ semiconductor and bleached shellac wax dielectric on plain aluminum gate electrode.

Two batches of transistors were prepared for each particular wax comprised in this study in combination with pentacene and C_60_, respectively. Considering that our fabrication mask consists of six positions for glass slides as the one depicted schematically in Fig. 10, and that the respective slide contains four transistors, a total of forty-eight OFETs was prepared for each wax and pentacene and likewise forty-eight OFETs for each wax and C_60_. While measuring the transistors we noticed, however, a significant discrepancy in the performance of the four OFETs on each slide, with the one at the bottom of the slide next to the MIM structure (*i.e.*, near the continuous electrode), denominated as OFET #4, performing always at a higher applied voltage than the one situated at the top of the slide, near the gate contact, *i.e.*, OFET #1 (see the schematic in Fig. 10). A systematic study of the thickness of the wax on the slide revealed that the wax deposited in a thicker layer at the bottom of the slide compared to the respective value at the top of the slide which represented the starting point of the blade sliding. We concluded that the time passed between the stop of the coating process to the moment the operator removed the excess material from the blade-sample interface represented a key factor in rendering more uniform films, since the solution material had a tendency to flow back into the already deposited film and to contribute to its increased thickness at the bottom of the electrode. This event was particularly exacerbated by (i) the low melting point of the waxes; (ii) the fast evaporation rate of the solvent (*i.e.*, chloroform for all the waxes except SWB for which *n*-octane was used); and (iii) the high tendency of the waxes to form solid films almost instantaneously after the evaporation of the solvent. As a consequence of this difficulty to control the deposition of the waxes, the OFETs functioned at various voltages, from 1 V to as high as 7–8 V, with the low voltage operating OFETs being in all cases the ones at positions #1 and #2 on the slides and the ones operating at higher voltages at positions #3 and #4 where the wax films were significantly thicker. As a consequence of this deposition non-uniformity, the measurement of the capacitance on the OFET slide did not accurately depict the capacitance of the entire film, since this value presented a gradient along the gate electrode. We measured the capacitance of the waxes by fabricating MIM devices with the top electrode deposited in various positions of the slide to mimic the positions of the S–D electrodes. The OFET fabrication resulted in functional devices in excess of 80% for all waxes employed in this study, but their performance was different even compared devices on the same slide. We present in the following Fig. 11–14 the results of champion devices for each combination of wax and organic semiconductors: pentacene (Fig. 11 and 12) and C_60_ (Fig. 13 and 14). Although the Fig. 11 and 12 on the one hand and Fig. 13 and 14 on the other hand belong together, we divided their representation in two figures each, in order to improve the clarity of the displayed data.

As can be seen from inspecting the graphs, the organic semiconductor C_60_ produced better results than pentacene, even though each of them had been purified three times by scrupulous vacuum sublimation, a step that proved to be crucial in the past in obtaining high performance devices.^72^ Although slight differences in operating voltages are visible for the waxes (mostly given by the working device being in a different position on the glass slide), as a general trend the operating voltage of C_60_ based devices was always lower than the respective value of pentacene based devices. This seems to be the general trend of hydrophobic dielectric surfaces as the six waxes analyzed here that offer better performance when interfaced with C_60_ rather than pentacene, a fact previously observed in our laboratory^74^. On the other hand, a more hydrophilic dielectric surface renders pentacene-based OFETs operation being superior to the one of C_60_-based OFETs.^73–76^ The set of Fig. 11–14 display on their left panel the transfer characteristics and on the right panel the respective output characteristics, with important information about the transistors and their performance being placed as insets on the transfer characteristics curves, for example specific capacitance, the calculated field effect mobility and the applied drain–source voltage, *V*_ds_ in each particular case. We demonstrate that operational devices are possible even with −10 mV applied *V*_ds_ for all the waxes and pentacene, and correspondingly +10 mV applied *V*_ds_ for BW, CW, LW, RBW and C_60_. The two shellac waxes functioned when interfaced to C_60_ only when the minimum voltage of +50 mV was applied to the drain–source terminals, but not lower (see Fig. 14).

Inspecting Fig. 11 and 12, one can see that applying an identical deposition recipe for the source–drain electrodes deposition as it was the case in this work, resulted in non-optimized deposition of the top contacts that translated in the existence of contact resistance for the combinations of BW, CW and the two shellac waxes with pentacene. This contact resistance is visible through the S-shape of the output curves at the low applied *V*_ds_ voltages in Fig. 11 and 12 and can be reduced by proper deposition procedure of the contact electrodes or functionalization of the underlying material, depending on the selected device geometry.^77,78^ On the other hand, it seems that the selected deposition technique of the contact electrodes for the C_60_-based OFETs is suitable for the generation of contact resistance free devices (see Fig. 13 and 14, the output panels).

**Fig. 15:**
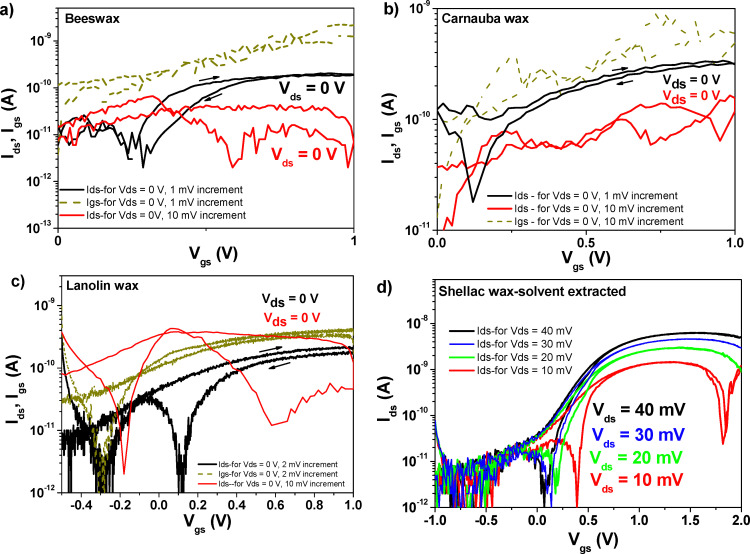
(a) Transfer characteristics of an OFET with beeswax dielectric and C_60_ semiconductor measured at zero volts drain–source voltage with various increment steps of the gate voltage displayed as insets in the graph; (b) transfer characteristics of an OFET with carnauba wax dielectric and C_60_ semiconductor measured at zero volts drain–source voltage with various increment steps of the gate voltage displayed as insets in the graph; (c) transfer characteristics of an OFET with lanolin wax dielectric and C_60_ semiconductor measured at zero volts drain–source voltage with various increment steps of the gate voltage displayed as insets in the graph; (d) investigation of the minimum possible source–drain voltage (*V*_ds_) able to raise the transfer characteristics of the OFET with shellac-solvent extracted dielectric and C_60_ semiconductor.

Fig. 15(a)–(c) presents OFET devices measured and presented already in Fig. 13, but this time with their lowest possible drain–source voltage which was still able to raise the transfer characteristics. We noticed that it is still possible to obtain the transfer characteristics (albeit with a very low drain–source current, *I*_ds_) even when the devices were measured very slowly with zero-volt *V*_ds_. Out of forty-eight devices containing the combination of each wax and C_60_ semiconductor, the number of OFETs that worked at zero *V*_ds_ applied voltage was three devices for beeswax, and two devices each for carnauba wax and lanolin wax. The rice bran wax device presented in Fig. 14(a) was the only one working with a zero-volt applied *V*_ds_, but was inadvertently destroyed during the recording of the recurrent measurement, and we will not show it here. However, as the panels (a)–(c) in Fig. 15 show, recording the transfer characteristics is possible only when measuring with 1 mV or 2 mV increment between measurement points; and it is not possible when the measurement increment is 10 mV, for example, or higher. This event is rather puzzling, and was observed in our previous work with caffeine dielectric and C_60_ semiconductor,^73^ but never recorded with pentacene semiconductor. In honesty, the source–drain (*I*_ds_) measured in the devices working with *V*_ds_ = 0 V is lower than the respective values of the leakage current through the dielectric (*i.e.*, the *I*_gs_) that is displayed as dark yellow dotted lines in the three graphs. This leakage current in Fig. 15 is in good agreement with the recorded values for the same samples measured and displayed in Fig. 13. Multiple explanations could account for the existence of this unexpected behavior (*i.e.*, the possibility to raise the transfer curve with 0 V for the *V*_ds_ voltage). One possibility could be the release of the surface charges trapped during the measurements done for recording the results presented in Fig. 13, since the curves in Fig. 15 were never observed for the first time measured (fresh) devices. Other mechanisms may play a role, too, like for example the slow discharge of the dielectric capacitor or even the existence of surface dipoles in the C_60_ semiconductor that could be recorded only for a very slowly applied voltage increment at the interface between the semiconductor to dielectric. Nevertheless, a systematic study of the process should shed more light into the rationale and occurrence of this unexpected phenomenon.

Fig. 15(d) presents the successive transfer measurements at various low *V*_ds_ voltages, showing that for the solvent extracted shellac wax and C_60_ based OFET, a voltage of 40 mV still cannot qualify as the minimum voltage able to raise the transfer characteristics, since at this voltage, the saturation line of the *I*_ds_ current still pitches down (for this particular sample, the minimum voltage that could be used to raise the transfer characteristic was 100 mV). This behavior is much more accentuated at progressively lower voltages, as the graph clearly demonstrates. For the SWE material, a device working at 1 V and displaying the minimum working voltage of 50 mV is presented in Fig. 13(e). A summary of the OFET parameters is presented in Table 7. Inspecting the individual graphs of Fig. 11–14 and the extracted OFET parameters in Table 7, is becomes obvious that the two most underperforming wax materials were the shellac wax obtained through the chemical bleaching process and the rice bran wax. For both materials, the devices recorded high leakage current, had low field effect mobility, and displayed pronounced hysteresis in their transfer characteristics compared to the other four waxes. The latter effect was to be expected in fact, after seeing the relaxation of the loss angle at low frequency in the impedance measurement of SWB or the very high loss angle (albeit without a visible relaxation) for the RBW, as the recorded impedance curves presented in Fig. 9 clearly demonstrate.

**Table 7:** OFET parameters of natural waxes on aluminum gate electrodes, with pentacene and C_60_ semiconductors

Wax material	OFET parameters											
	With pentacene						With C_60_					
	*C*_0d_ (nF cm^−2^)	*V*_th_ (V)	Lowest *V*_ds_ possible (mV)	*I*_ON_/*I*_OFF_	*μ* (cm^2^ V^−1^ s^−1^)	*S*_SW_ (mV dec^−1^)	*C*_0d_ (nF cm^−2^)	*V*_th_ (V)	Lowest *V*_ds_ possible (mV)	*I*_ON_/*I*_OFF_	*μ* (cm^2^ V^−1^ s^−1^)	*S*_*SW*_ (mV dec^−1^)
BW	81.5	−0.77	−10	4.15 × 10^4^	6 × 10^−2^	75	131.2	0.1	0	2154	4 × 10^−2^	200
CW	126.3	−0.83	−10	2.77 × 10^4^	0.35	130	128.7	−0.12	0	106	0.2	280
LW	354.3	−0.41	−10	1.12 × 10^4^	0.1	160	368.4	−0.15	0	79	0.13	415
RBW	123.2	−2.53	−10	6.28 × 10^4^	3 × 10^−2^	308	115.3	0.85	0	1185	4 × 10^−2^	165
SWE	242.5	−0.79	−10	3.34 × 10^4^	6 × 10^−2^	133	243.1	0.22	+50	1323	7 × 10^−3^	115
SWB	233.7	−1.15	−10	3.66 × 10^4^	7 × 10^−2^	115	265.8	0.75	+50	111	2 × 10^−3^	1025

The presence of ionic impurities in the dielectric has indeed an influence on the performance of the organic field effect transistors, as it was demonstrated in our previous work on polyvinyl alcohol.^71^ However, in the present study mostly the SWB dielectric seem to have this problem more pronounced, probably due to its own fabrication history. Indeed, it is possible that, the presence of remnant solvents in the two wax samples following their extraction processes from the parent shellac resin (in case of SWB) or the separation itself from the rice bran oil in case of RBW, rendered the lowest performance of the two among the investigated waxes. However, both events are not necessarily bad news, since (1) in the case of shellac wax, the wax can be extracted through a more efficient and “green” solvent extraction process, so in effect there exists a viable alternative to produce better quality shellac wax that in fact we have already presented here; and (2) rice crops should definitely not be employed for electronics fabrications through the extraction of rice bran wax from rice bran oil, but rather directed for food consumption in a world of constantly increasing population.

## 4. Conclusions

In this work, we thoroughly analyzed six different waxes of plant and animal origin and investigated their performance as dielectrics for the fabrication of field effect transistors. Such materials containing mostly triglycerides are generally known to be biodegradable under aerobic environmental conditions. Microorganisms present in soil and compost environments can enzymatically break down these natural waxes into simpler compounds such as fatty acids, which are further metabolized into carbon dioxide, water, and biomass. The rate of biodegradation may vary, depending on the wax composition and environmental conditions (*e.g.*, temperature, microbial activity, moisture), but overall, these materials are classified as biodegradable.^79–81^ Being hydrophobic materials, these waxes could be interfaced with H-bonded semiconductors for the fabrication of benign electronics.^12,13,82–86^ We demonstrated that deposition of these waxes in thin layers is possible *via* a low-cost laboratory technique (blade coating), and functional organic field effect transistor devices could be obtained with virtual ease, albeit with a relative non-uniformity of their operation window, dictated by the particular solidification route of each solution processed wax. The device variability that we encountered when fabricating OFETs with dielectric materials originating from solutions of highly volatile solvents as chloroform or *n*-octane, can be mitigated by employing larger substrates for the fabrication process when the OFETs are aligned on the same gate electrode as it was the case of this study. In that case the transistors placed at the end of the gate, where the blade coating process ends, could be discarded from the study, and the statistics of the results would be more uniform across the transistors situated in the middle of the coating process. The devices presented here represent the highlights of our entire investigation, showing that operating voltages as low as 1 V are possible for the scanned window, and the respective devices function properly even with ±10 mV applied drain–source voltage. In some particular cases, champion devices fabricated with natural waxes and C_60_ semiconductor function even with 0 V applied voltage between source and drain terminals. In addition to their low voltage operation, the subthreshold swing values in the range of 100 mV dec.^−1^ or lower are among the best organic field effect transistors devices ever reported,^87–89^ keeping in mind that values ∼70 mV dec.^−1^ are found in highly optimized MOSFET devices.^90^ The material scientists worldwide are actively searching for bio-degradable, bio-origin and environmentally nontoxic substrates, dielectrics, semiconductors and conductors, for many different applications. These natural waxes are very good candidates for dielectrics and packaging substrates, which have a nontoxic, sustainable footprint. Future consumable electronics will definitely need abundant and sustainable materials like these benign biomaterials.

## Conflicts of interest

The authors declare no conflicts of interest.

## Data Availability

Data are available upon request from the authors.

## References

[cit1] Stadlober B., Zirkl M., Irimia-Vladu M. (2019). Chem. Soc. Rev..

[cit2] Hultman L., Mazur S., Ankarcrona C., Palmqvist A., Abrahamsson M., Antti M.-L., Baltzar M., Bergström L., de Laval P., Edman L., Erhart P., Kloo L., Lundberg M. W., Mikkelsen A., Moons E., Persson C., Rensmo H., Rosén J., Rudén C., Selleby M., Sundgren J.-E., Dick Thelander K., Tybrandt K., Weihed P., Zou X., Åstrand M., Platzer Björkman C., Schneider J. M., Eriksson O., Berggren M. (2024). Nat. Mater..

[cit3] Brasier N., Wang J., Gao W., Sempionatto J. R., Dincer C., Ates H. C., Güder F., Olenik S., Schauwecker I., Schaffarczyk D., Vayena E., Ritz N., Weisser M., Mtenga S., Ghaffari R., Rogers J. A., Goldhahn J. (2024). Nature.

[cit4] Oldroyd P., Velasco-Bosom S., Bidinger S. L., Hasan T., Boys A. J., Malliaras G. G. (2025). Nat. Prot..

[cit5] Ge S., Nemiroski A., Mirica K. A., Mace C. R., Hennek J. W., Kumar A. A., Whitesides G. M. (2020). Angew. Chem., Int. Ed..

[cit6] Nie Z., Kwak J. W., Han M., Rogers J. A. (2024). Adv. Mater..

[cit7] Cho S., Shaban S. M., Song R., Zhang H., Yang D., Kim M.-J., Xiong Y., Li X., Madsen K., Wapnick S., Zhang S., Chen Z., Kim J., Guinto G., Li M., Lee M., Nuxoll R. F., Shajari S., Wang J., Son S., Shin J., Aranyosi A. J., Wright D. E., Kim T.-I., Ghaffari R., Huang Y., Kim D.-H., Rogers J. A. (2024). Sci. Transl. Med..

[cit8] Zhong D., Nishio Y., Wu C., Jiang Y., Wang W., Yuan Y., Yao Y., Tok J. B.-H., Bao Z. (2024). ACS Nano.

[cit9] McCoy R., Wang K., Treiber J., Fu Y., Malliaras G. G., Salleo A., Owens R. M. (2025). J. Mater. Chem. B.

[cit10] Camus A., Choe S., Bour-Cardinal C., Isasmendi J., Cho Y., Kim Y., Irimia C. V., Yumusak C., Irimia-Vladu M., Rho D., Myung J., Santato C. (2024). Commun. Mater..

[cit11] Radovanović M. R., Popović Ž., Kojić S., Piper D., Luzio A., Bonacchini G. E., Caironi M., Stojanović G. M. (2025). Adv. Eng. Mater..

[cit12] Irimia-Vladu M., Kanbur Y., Camaioni F., Yumusak C., Vlad A. A., Irimia C. V., Operamolla A., Farinola G., Romanazzi G., Suranna G. P., González N., Molina M. C., Bautista L. F., Langhals H., Glowacki E. D., Sariciftci N. S. (2019). Chem. Mater..

[cit13] Glowacki E. D., Irimia-Vladu M., Kaltenbrunner M., Gąsiorowski J., White M. S., Romanazzi G., Suranna G. P., Mastrorilli P., Sekitani T., Bauer S., Someya T., Torsi L., Sariciftci N. S. (2013). Adv. Mater..

[cit14] Yokota T., Kuribara K., Tokuhara T., Zschieschang U., Klauk H., Takimiya K., Sadamitsu Y., Hamada M., Sekitani T., Someya T. (2013). Adv. Mater..

[cit15] Feron K., Lim R., Sherwood C., Keynes A., Brichta A., Dastoor P. C. (2018). Int. J. Mol. Sci..

[cit16] Bolognesi M. , Prosa M. and Seri M., in Sustainable Strategies in Organic Electronics, ed. A. Marrocchi, Woodhead Publishing Series in Electronic and Optical Materials, Woodhead Publishing, 2022, pp. 297–338

[cit17] Irimia-Vladu M. (2014). Chem. Soc. Rev..

[cit18] Cetkovic A., Bellapianta A., Irimia-Vladu M., Hofinger J., Yumusak C., Corna A., Scharber M. C., Zeck G., Sariciftci N. S., Bolz M., Salti A. (2022). Int. J. Mol. Sci..

[cit19] Park J., Lee Y., Kim T. Y., Hwang S., Seo J. (2022). ACS Appl. Electron. Mater..

[cit20] Cao Y., Uhrich K. E. (2018). J. Bioact. Compat. Polym..

[cit21] Chauhan A. K., Jha P., Aswal D. K., Yakhmi J. V. (2022). J. Electron. Mater..

[cit22] Trudel S. (2023). Phys. Today.

[cit23] Wu X., Ma Y., Zhang G., Chu Y., Du J., Zhang Y., Li Z., Duan Y., Fan Z., Huang J. (2015). Adv. Funct. Mater..

[cit24] Sun L., Diaz-Fernandez Y. A., Gschneidtner T. A., Westerlund F., Lara-Avilab S., Moth-Poulsen K. (2014). Chem. Soc. Rev..

[cit25] Someya T., Bao Z., Malliaras G. (2016). Nature.

[cit26] Muhl S., Beyer B. (2014). Electronics.

[cit27] Wang L., Chen D., Jiang K., Shen G. (2017). Chem. Soc. Rev..

[cit28] Stadlober B., Karner E., Petritz A., Fian A., Irimia-Vladu M. (2015). IEEE J. Solid-State Circuits.

[cit29] Baumgartner M. , Coppola M. E., Sariciftci N. S., Głowacki E. D., Bauer S. and Irimia-Vladu M., Emerging “Green” Materials and Technologies for Electronics, in Green Materials for Electronics, ed. M. Irimia-Vladu, E. D. Glowacki, N. S. Sariciftci, S. Bauer, Wiley-VCH, 2017

[cit30] Saadi D., Yumusak C., Zrinski I., Mardare A. I., Romdhane S., Sariciftci N. S., Irimia-Vladu M., Scharber M. C. (2023). Phys. Status Solidi A.

[cit31] Jiang S., Feng P., Yang Y., Du P., Shi Y., Wan Q. (2016). IEEE Electron Device Lett..

[cit32] Wang L., Wang K., Lou Z., Jiang K., Shen G. (2018). Adv. Funct. Mater..

[cit33] Diemer P. J., Harper A. F., Niazi M. R., Petty II A. J., Anthony J. E., Amassian A., Jurchescu O. D. (2017). Adv. Mater. Technol..

[cit34] Tulloch A. P. (1980). Bee World.

[cit35] Wolfmeier U. , Schmidt H., Heinrichs F.-L., Michalczyk G., Payer W., Dietsche W., Boehlke K., Hohner G. and Wildgruber J., “Waxes”, Ullmann's Encyclopedia of Industrial Chemistry, Wiley-VCH, Weinheim, 2000, vol. 39, pp. 112–172

[cit36] Krendlinger E. , Wolfmeier U., Schmidt H., Heinrichs F.-L., Michalczyk G., Payer W., Dietsche W., Boehlke K., Hohner G. and Wildgruber J., “Waxes”, Ullmann's Encyclopedia of Industrial Chemistry, Wiley-VCH, Weinheim, 2015, pp. 1–63

[cit37] EFSA Panel on Food Additives and Nutrient Sources added to Food (ANS) (2012). EFSA J..

[cit38] Barnett G. A. (1986). Cosmet. Toiletries.

[cit39] Hoppe U. , The lanolin book, ed. P. Beiersdorf, 1999, vol. **3**, pp. 1–285

[cit40] Riemenschneider W. and Bolt H. M., “Esters, Organic”, Ullmann's Encyclopedia of Industrial Chemistry, 2005, vol. 13, pp. 245–266

[cit41] The Merck Index-An Encyclopedia of Chemicals, Drugs, and Biologicals, Whitehouse Station, ed. M. J. O’Neil, Merck and Co., Inc., NJ, 2006, p. 929

[cit42] Zirwas M. J., Stechschulte S. A. (2008). J. Clin. Aesthet. Dermatol..

[cit43] Yoon S. H., Rhee J. S. (1982). J. Am. Oil Chem. Soc..

[cit44] Abhirami P., Modupalli N., Natarajan V. (2020). J. Food Process Preserv..

[cit45] Orthoefer F. T. , Rice Bran Oil, in Bailey's Industrial Oil and Fat Products, ed. F. Shahidi, John Wiley & Sons, 6th edn, 2005, vol. 2, p. 465

[cit46] Kittipongpatana O. S., Trisopon K., Wattanaarsakit P., Kittipongpatana N. (2024). Pharmaceutics.

[cit47] Irimia-Vladu M., Głowacki E. D., Schwabegger G., Leonat L., Akpinar H. Z., Sitter H., Bauer S., Sariciftci N. S. (2013). Green Chem..

[cit48] Kim S., Yumusak C., Irimia C. V., Bednorz M., Yenel E., Kus M., Sariciftci N. S., Shim B. S., Irimia-Vladu M. (2023). Turk. J. Chem..

[cit49] Skaf D., Carneiro Gomes T., Hussein R. N., Nagesh G., Ahamed M. J., Carmichael T. B., Rondeau-Gagné S. (2024). ACS Appl. Polym. Mater..

[cit50] Skaf D., Carneiro Gomes T., Majidzadeh R., Hussein R. N., Carmichael T. B., Rondeau-Gagné S. (2023). Flexible Printed Electron..

[cit51] Baek S. W., Ha J.-W., Yoon M., Hwang D.-H., Lee J. (2018). ACS Appl. Mater. Interfaces.

[cit52] Irimia-Vladu M., Sariciftci N. S. (2024). Polym. Intern..

[cit53] Won S. M., Koo J., Crawford K. E., Mickle A. D., Xue Y., Min S., McIlvried L. A., Yan Y., Kim S. B., Lee S. M., Kim B. H., Jang H., MacEwan M. R., Huang Y., Gereau IV R. W., Rogers J. A. (2018). Adv. Funct. Mater..

[cit54] Argyropoulos D. S., Pajer N., Crestini C. (2021). JoVE.

[cit55] Nečas D., Klapetek P. (2012). Open Phys..

[cit56] Duce C., Orsini S., Spepi A., Colombini M. P., Tiné M. R., Ribechini E. (2015). J. Anal. Appl. Pyrolysis.

[cit57] Craig R. G., Powers J. M., Peyton F. A. (1971). J. Dent. Res..

[cit58] Gigante V., Cinelli P., Righetti M. C., Sandroni M., Polacco G., Seggiani M., Lazzeri A. (2020). Polymers.

[cit59] Al-Gousous J., Penning M., Langguth P. (2015). Int. J. Pharm..

[cit60] Barik A., Patnaik T., Parhi1 P., Swain S. K., Dey R. K. (2017). Polym. Bull..

[cit61] Rudra Murthy B. V., Thanaiah K., Gumtapure V. (2022). Sol. Energy.

[cit62] Basson I., Reynhardt E. C. (1988). J. Phys. D: Appl. Phys..

[cit63] Bergamonti L., Cirlini M., Graiff C., Lottici P. P., Palla G., Casoli A. (2022). Appl. Sci..

[cit64] Svečnjak L., Chesson L. A., Gallina A., Maia M., Martinello M., Mutinelli F., Muzf M. N., Nunes F. M., Saucy F., Tipple B. J., Wallner K., Was E., Waters T. A. (2019). J. Apic. Res..

[cit65] Vandenburg L. E., Wilder E. A. (1967). J. Am. Oil Chem. Soc..

[cit66] Yumusak C., Mayr F., Wielend D., Kahraman B., Kanbur Y., Langhals H., Irimia-Vladu M. (2022). Isr. J. Chem..

[cit67] Udum Y., Denk P., Workneh G. A., Apaydin D. H., Nevosad A., Teichert C., White M. S., Sariciftci N. S., Scharber M. C. (2014). Org. Electron..

[cit68] Macdonald J. R. , Impedance Spectroscopy: Emphasizing Solid Materials and Systems, Wiley, 1987

[cit69] Irimia-Vladu M., Fergus J. W. (2006). Synth Met..

[cit70] Alami A. H., Aokal K., Zhang D., Taieb A., Faraj M., Alhammadi A., Soudan B., El-Hajjar J., Irimia-Vladu M. (2019). Int. J. Energy Res..

[cit71] Egginger M., Irimia-Vladu M., Schwödiauer R., Tanda A., Frischauf I., Bauer S., Sariciftci N. S. (2008). Adv. Mater..

[cit72] Yumusak C., Sariciftci N. S., Irimia-Vladu M. (2020). Mater. Chem. Front..

[cit73] Irimia C. V., Yumusak C., Ban B., Leeb E., Mayr F., Schimanofsky C., Mardare A. I., Molnar M. A., Teichert C., Sariciftci N. S., Irimia-Vladu M. (2025). IEEE J. Flex. Electon..

[cit74] Coppola M. E., Petritz A., Irimia C. V., Yumusak C., Mayr F., Bednorz M., Matkovic A., Aslam M. A., Saller K., Schwarzinger C., Ionita M. D., Schiek M., Smeds A. I., Salinas Y., Brüggemann O., D’Orsi R., Mattonai M., Ribechini E., Operamolla A., Teichert C., Xu C., Stadlober B., Sariciftci N. S., Irimia-Vladu M. (2023). Glob. Chall..

[cit75] D’Orsi R., Irimia C. V., Lucejko J. J., Kahraman B., Kanbur Y., Yumusak C., Babudri F., Irimia Vladu M., Operamolla A. (2022). Adv. Sustainable Syst..

[cit76] Ivić J., Petritz A., Irimia C. V., Kahraman B., Kanbur Y., Bednorz M., Yumusak C., Aslam M. A., Matković A., Saller K., Schwarzinger C., Schühly W., Smeds A. I., Salinas Y., Schiek M., Mayr F., Xu C., Teichert C., Osiac M., Sariciftci N. S., Stadlober B., Irimia-Vladu M. (2022). Adv. Sust. Syst..

[cit77] Stadlober B., Haas U., Gold H., Haase A., Jakopic G., Leising G., Koch N., Rentenberger S., Zojer E. (2007). Adv. Funct. Mater..

[cit78] Petritz A., Krammer M., Sauter E., Gärtner M., Nascimbeni G., Schrode B., Fian A., Gold H., Cojocaru A., Karner-Petritz E., Resel R., Terfort A., Zojer E., Zharnikov M., Zojer K., Stadlober B. (2018). Adv. Funct. Mater..

[cit79] Modupalli N., Natarajan V. (2022). Pharma Innov. J..

[cit80] Arunkumar N., Gulsar Banu J., Gopalakrishnan N., Prakash A. H. (2017). Int. J. Curr. Microbiol. App. Sci..

[cit81] Hanstveit A. O. (1992). Chemosphere.

[cit82] Koehler M., Farka D., Yumusak C., Sariciftci N. S., Hinterdorfer P. (2020). ChemPhysChem.

[cit83] Irimia-Vladu M., Troshin P. A., Reisinger M., Shmygleva L., Kanbur Y., Schwabegger G., Bodea M., Schwödiauer R., Mumyatov A., Fergus J. W., Razumov V. F., Sitter H., Sariciftci N. S., Bauer S. (2010). Adv. Funct. Mater..

[cit84] Irimia-Vladu M., Glowacki E. D., Troshin P. A., Susarova D. K., Krystal O., Schwabegger G., Ullah M., Kanbur Y., Bodea M. A., Razumov V. F., Sitter H., Bauer S., Sariciftci N. S. (2012). Adv. Mater..

[cit85] Kahraman B., Yumusak C., Mayr F., Wielend D., Kotwica K., Irimia C. V., Leeb E., Cobet M., Sariciftci N. S., Irimia-Vladu M. (2024). J. Mater. Chem. C.

[cit86] Kanbur Y., Coskun H., Głowacki E. D., Irimia-Vladu M., Sariciftci N. S., Yumusak C. (2019). Org. Electron..

[cit87] Newman C. R., Frisbie C. D., da Silva Filho D. A., Bredas J.-L., Ewbank P. C., Mann K. R. (2004). Chem. Mater..

[cit88] Klauk H. (2018). Adv. Electron. Mater..

[cit89] Bisoyi S., Zschieschang U., Kang M. J., Takimiya K., Klauk H., Tiwari S. P. (2014). Org. Electron..

[cit90] Sze S. M. , in Physics of Semiconductor Devices, ed. K. K. Ng, Wiley, New York, 3rd edn, 2007, ch. 6.2.4, p. 315

